# A systematic review of the mental health changes of children and young people before and during the COVID-19 pandemic

**DOI:** 10.1007/s00787-022-02060-0

**Published:** 2022-08-12

**Authors:** Laura Kauhanen, Wan Mohd Azam Wan Mohd Yunus, Lotta Lempinen, Kirsi Peltonen, David Gyllenberg, Kaisa Mishina, Sonja Gilbert, Kalpana Bastola, June S. L. Brown, Andre Sourander

**Affiliations:** 1 Research Centre for Child Psychiatry, University of Turku, Turku, Finland; 2 INVEST Research Flagship, University of Turku, Turku, Finland; 3 Department of Psychology, Faculty of Social Sciences and Humanities, Universiti Teknologi Malaysia, Johor, Malaysia; 4 National Institute for Health and Welfare, Helsinki, Finland; 5 Department of Adolescent Psychiatry, Helsinki University Central Hospital, Helsinki, Finland; 6 Department of Psychology, Institute of Psychiatry, Psychology & Neuroscience (IoPPN), King’s College London, England London, UK; 7 Department of Child Psychiatry, Turku University Hospital, Turku, Finland

**Keywords:** COVID-19 pandemic, Children, Adolescent, Mental health, Young people

## Abstract

There is increasing knowledge that the COVID-19 pandemic has had an impact on mental health of children and young people. However, the global evidence of mental health changes before compared to during the COVID-19 pandemic focusing on children and young people has not been systematically reviewed. This systematic review examined longitudinal and repeated cross-sectional studies comparing before and during COVID-19 pandemic data to determine whether the mental health of children and young people had changed before and during the COVID-19 pandemic. The Web of Science, PubMed, Embase and PsycINFO databases were searched to identify peer-reviewed studies that had been published in English and focused on children and young people between 0 and 24 years of age. This identified 21 studies from 11 countries, covering more than 96,000 subjects from 3 to 24 years of age. Pre-pandemic and pandemic data were compared. Most studies reported longitudinal deterioration in the mental health of adolescents and young people, with increased depression, anxiety and psychological distress after the pandemic started. Other findings included deteriorated negative affect, mental well-being and increased loneliness. Comparing data for pandemic and pre-pandemic periods showed that the COVID-19 pandemic may negatively impact the mental health of children and young people. There is an urgent need for high-quality research to address the impact, risks and protective factors of the pandemic on their mental health, as this will provide a good foundation for dealing with future health emergencies and other crises.

**Supplementary Information:**

The online version contains supplementary material available at 10.1007/s00787-022-02060-0.

## Introduction

There is growing concern about the negative effects that the COVID-19 pandemic is having on the mental health of children and young people, particularly those who are already vulnerable. A number of factors have contributed to mental health issues in these age groups, including emotional, physiological and behavioral stress. These have been due to factors such as social isolation, due to school closures, parental stress about the virus and employment, increases in undetected child abuse, increased cyber bullying, due to more online activities, and the trauma of losing family members [1, 2]. In addition, the COVID-19 pandemic may have increased loneliness, depression and anxiety and decreased life satisfaction [3]. Increased suicidality and suicide attempts by adolescents during the pandemic has also been reported [4]. Lockdowns and physical distancing may have had a different impact on some children and young people, depending on their age, gender, ethnicity, family circumstances, socioeconomic situation and any pre-existing mental health problems [5–8].

To our knowledge, five systematic reviews have been published on the mental health of children and young people during the COVID-19 pandemic, four in 2021 and one in 2020. Meherali et al. and Nearchou et al. only included cross-sectional studies carried out at one time-point [9, 10]. Racine et al. conducted a meta-analysis, which reported that the global prevalence of anxiety and depression among children and adolescent were 25.2% and 20.5% with 95% confidence intervals (CI) of 21.2–29.7 and 17.2–24.4, respectively, for the period from February to July 2020 [11]. Samji et al. attempted a broader systematic review, by examining 116 papers with any empirical study design [12], while Panchal et al. systematic review included 61 papers [13]. However, more than 70% included studies in both reviews were cross-sectional studies and they also included specific clinical subgroups. This makes it difficult to discern the mental health changes before and during the COVID-19 pandemic toward the general children and young people population samples. Samji et al. have stated that the priority for COVID-19 research should be representative samples and/or longitudinal follow-up studies that have the potential to demonstrate changes in mental health symptoms before and after the pandemic [12].

Different indicators for mental health problems should be measured repeatedly before and during the COVID-19 pandemic to assess changes in mental health trends during the pandemic. These should use directly comparable measures, sampling designs and convergent information from multiple sources, such as parents, teachers and children [14, 15].

The aim of this study was to conduct a systematic review of the existing global literature, so that we could compare the mental health of children and young people aged 0–24, before and during the COVID-19 pandemic, and identify any changes.

## Materials and methods

### Search strategy

The review study followed the Preferred Reporting Items for Systematic Reviews and Meta-Analyses (PRISMA) guidelines [16]. The protocol of the review was prospectively registered with the International Prospective Register of Systematic Reviews (PROSPERO number: CRD42021238999). The Web of Science, PubMed, Embase and PsycINFO electronic databases were used to identify peer-reviewed papers published in English from 1 January 2020 to 22 March 2021. The search syntax for each database is listed in Table S1 in the Supplementary Appendix. We also used the backward snowballing technique to search for other relevant papers [17], by looking at the reference lists of the selected papers.

### Inclusion and exclusion criteria

Studies with children and young people aged 0–24 years were included. Children were defined as 0–9 years old and young people as 10–24 years of age. We refer to the definition of adolescents (10–19 years old) and young people (10–24 years old) by the World Health Organization [18, 19] that include adolescents as defined by Sawyer et al. (10–24 years old) [20]. Population, community or school-based studies were included. We did not include studies that focused on individuals aged 25 or older or studies that did not report the specific age ranges of the subjects.

The review included any observational studies that used surveys or interviews to determine the mental health symptoms of subjects before and during the pandemic. These included longitudinal, repeated cross-sectional, cohort, panel, time series and time trend studies. We excluded single cross-sectional studies, studies that using modeling to predict the impact of the pandemic on mental health or studies that used different measures to compare outcomes before and during the pandemic. The periods before and during the pandemic were the pandemic periods defined by the authors of the included studies and at least one corresponding pre-pandemic period. It is worth noting that we have previously reported findings on the registered mental health service use, self-harm and suicides based on administrative data [21] so these types of studies were also excluded in this review.

The following mental health outcomes were included; externalizing and internalizing problems, attention-deficit/hyperactivity problems, negative and positive effects, depression, anxiety, psychological distress, health-related quality of life, peer problems and loneliness. Only studies that reported at least one validated measure of these mental health outcomes before and during the COVID-19 pandemic were included. The studies were only included if the same measures and/or methods were used for each outcome before and during the pandemic.

### Study selection and retrieval process

The screening and study selection processes were independently conducted by two reviewers (LK and WMAWMY), first based on the titles and abstracts, after removing the duplicates. If there was insufficient information in the title and abstracts, the full texts were retrieved and reviewed to determine the study's eligibility. Any disagreements were discussed with a senior researcher (DG) and professor (AS). The two reviewers independently conducted full-text assessments based on the predefined inclusion and exclusion criteria. Both reviewers cross-checked the included papers and any disagreements were discussed, and resolved, with the senior researcher and professor.

### Quality assessment

The quality of the studies was assessed using 14 items from the National Institutes for Health (NIH) Study Quality Assessment Tool for Observational Cohort and Cross-Sectional Studies [22]. These covered the study power, the strength of the causality in the association between exposure and outcomes and the risks of potential selection bias, information bias, measurement bias or confounding bias. Studies were then categorized as good, fair or poor quality and reported using the total score for each study (Table S2 in the Supplementary Appendix).

### Data extraction and synthesis

We then extracted the relevant data and placed them into an Excel spreadsheet, version 16.43 (Microsoft Corp, Redmond, WA, USA). These included the author, country, study design, sample, informants, age, sampling methods and response rates. We also extracted the pre-pandemic and pandemic timepoints, mental health outcomes and measures, the descriptive data before and during the pandemic and the inferential analysis results. The key findings were extracted and the changes to mental health were divided according to each outcome into three categories that showed whether the subject’s mental health had deteriorated, improved or showed no difference. Where available, we also extracted information on the influence on gender, age and existing poor mental health problems and whether these were significant factors in the association to mental health before and during the pandemic.

## Results

### Study selection and retrieval processes

**Fig. 1:**
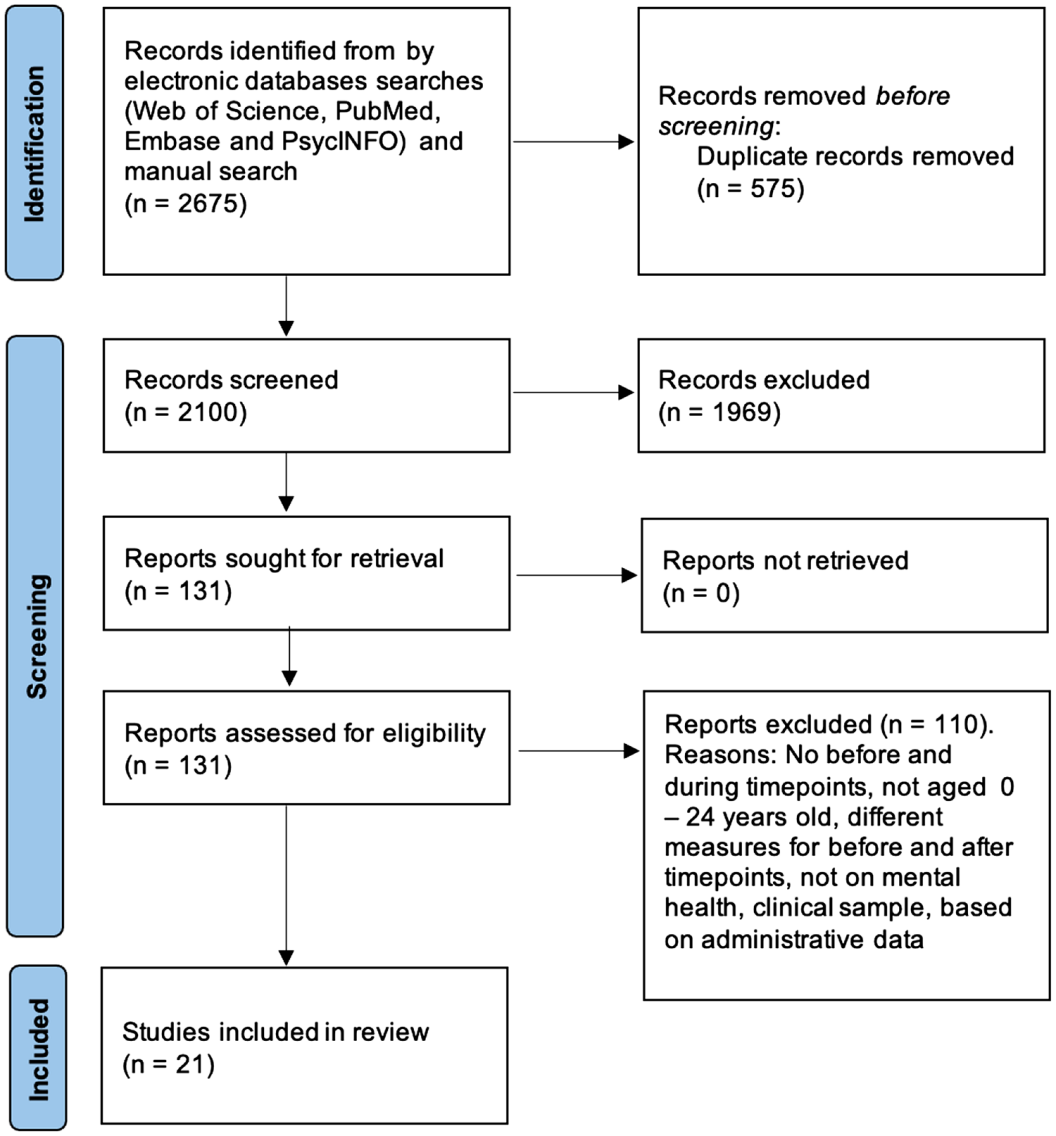
PRISMA Flow diagram

The electronic and manual searches identified 2675 citations. A total of 575 duplicates were removed and the other 2100 records were screened for eligibility. Following the title and abstract screening, 1969 records were excluded and the full texts of 131 papers were evaluated. We excluded 110 full texts because they did not compare before and during COVID-19 pandemic timepoints, they did not provide specific data for individuals aged 0–24, used different measure before and during pandemic timepoints, focus on clinical samples or were based on administrative data. At the end of this process, 21 studies were included in the qualitative synthesis. Figure 1 shows the PRISMA flow diagram for the screening and study selection processes.

### Description of the included studies

The systematic review comprised 21 mental health studies published in 2020 and 2021, covering a total study population of more than 96,000 subjects aged from 3 to 24 years of age in 11 countries. The summary of the studies and the mental health changes are presented in Table 1. The data ranged from January 2009, before the pandemic, to October 2020, during the pandemic. Twelve studies collected data during the initial phase of the pandemic (up to April 2020) [8, 23–33], seven studies collected data between April and June 2020 [7, 34–39], and one study each collected data from June to July 2020 [40] and October 2020 [41] respectively. Five studies were conducted in the UK [24, 27, 28, 35, 40] and in China [23, 30–33], two studies in the USA [8, 25], Italy [26, 36] and Australia [29, 39] and one study in India [34], Canada [37], Spain [38], Germany [7] and Iceland [41]. The NIH rating showed that 12 were rated as good-quality studies and nine studies were rated as fair quality (Table S2 in the Supplementary Appendix).

**Table 1:** Summary of included studies

Author, year, country	Sample, age, informants and response rates	Before and during pandemic time points	Outcome(s) and measure(s)	Key findings	Before/during data (mean ± SD otherwise stated), Changes analysis (eg. statistical significance, effect sizes)	Direction of mental health changes	Mental health changes based on age, gender or existing poor mental health
Repeated cross-sectional							
Chen et al. (2021), China [33]	Snowball sampling using smart phone-based WeChat-Wenjuanxing. Self-reports from students aged 11–20 in all 34 Chinese provinces before pandemic: 9554. During: 3886 (no response rates stated)	February 2020 vs April 2020	(1) Depression (Center for Epidemiological Studies Depression scale) (2) Anxiety (Generalized Anxiety Disorder 7 scale)	Increased depression and anxiety symptoms during the pandemic	(1) Depression (2) Anxiety; Before: (1) 14.06 ± 10.80, (2) 2.28 ± 3.54; During: (1) 19.29 ± 11.82, (2) 3.98 ± 4.46; Changes: (1) *p* < 0.001, (2) *p* < 0.001	(1) Depression deteriorated (2) Anxiety deteriorated	Gender: increased risk of depression and anxiety among female; Age: increased risk of depression and anxiety among senior secondary school (age 16–20)
Gray et al. (2020), Wales (UK) [40]	Online snowball sampling, advertised via social and mass media. 2019 data from on those aged 16–24 from large-scale, self-reported National Survey for Wales. Before pandemic: not stated. During: 703 (no subpopulation response rates stated)	April 2018 to March 2019 vs June and July 2020	Well-being (Warwick-Edinburgh Mental Well-being scale)	Large decrease in psychological well-being during pandemic	Mental well-being; Before: 50.3; During: 41.2; Changes: *p* < 0.001, Hedge’s g 0.95, 95% CI 0.83–1.06	Mental well-being deteriorated	Age: greater increase in psychological distress among young people (18–24) compared to older general population
Liébana-Presa et al. (2020), Spain [38]	Two self-reported cross-sectional samples obtained by convenience, non-probability sampling of students aged 13–17 from two schools with similar socio-demographic characteristics in a Spanish town. Before pandemic: 202. During: 98 (response rates during 36%)	February 2020 vs May 2020	(1) Emotional stress (2) Physiological stress (3) Behavioral stress (all subscales of Student Stress Inventory-Stress Manifestations)	Statistically significant and higher manifestation of emotional stress during pandemic	Stress: (1) Emotional (2) Physiological (3) Behavior; Before: (1) 21.02 ± 8.88, (2) 24.63 ± 9.83, (3) 9.38 ± 4.16; During: (1) 24.63 ± 9.83, (2) 10.15 ± 4.54, (3) 9.95 ± 4.21; Changes: (1) *p* < 0.05, (2) *p* = 0.340, (3) *p* = 0.266	(1) Emotional stress deteriorated (2) No significant change (physiological stress) deteriorated (3) No significant change (behavioral stress)	N/A
Ravens‐Sieberer et al. (2021), Germany [7]	Pandemic population-based representative online survey Corona and Psyche (COPSY) compared with pre-pandemic data from nationwide, longitudinal, representative BELLA cohort study Before: 1556. During: 1586 families with adolescents aged 7–17 years. Self-reported and/or parent-reported COPSY response rate 45.8%. No response rate for BELLA	BELLA 2017 vs COPSY May and June 2020	(1) Quality of life (KIDSCREEN-10 index) (2) Depression (German version of the Center for Epidemiological Studies Depression scale) (3) Anxiety (Screen for Child Anxiety Related Disorders (4) Mental health (5) Emotional symptoms (6) Conduct problems (7) Hyperactivity (8) Peer problems (4–8 all Strengths and Difficulties Questionnaire)	Adolescents experienced significantly lower quality of life, more mental health problems (anxiety), conduct problems, hyperactivity, and peer problems during the pandemic	(1)Quality of life (2) Depression (3) Anxiety (4) Mental health (5) Emotional (6) Conduct (7) Hyperactivity (8) Peer problems; Before (%): low, normal/high: (1) 15.3, 84.7 high: (2) not available, (3) 14.9% normal, borderline, abnormal: (4) 82.4, 7.8, 9.9, (5) 83.6, 6.2, 10.2, (6) 86.9, 6.5, 6.6, (7) 87.2, 5.1, 7.7, (8) 88.6, 3.9, 7.5; During (%): low, normal/high (1) 40.2, 59.8 high: (2) Not available, (3) 24.1% normal, borderline, abnormal: (4) 69.6, 12.5, 17.8, (5) 79.0, 7.7, 13.3, (6) 80.8, 9.1, 10.0, (7) 76.4, 8.9, 14.6, (8) 78.2, 10.2, 11.5; Changes: (1) *p* < 0.001, (2) *p* > 0.05, (3) *p* < 0.001, (4) *p* < 0.001, (5) *p* = 0.007, (6) *p* < 0.001, (7) *p* < 0.001, (8) *p* < 0.001	(1) Quality of life deteriorated (2) No significant change in depression, (3) mental health (anxiety) deteriorated (4) mental health (total mental health) deteriorated (5) no significant change in emotional symptoms (6) mental health (conduct problems) deteriorated (7) Mental health (hyperactivity) deteriorated (8) mental health (peer problems) deteriorated	Age: higher increases in mental health problems among boys (parental report 7–17)
Longitudinal							
Alivernini et al. (2020), Italy [36]	Adolescents aged 14–19 from several different geographical areas of Italy who participated in a self-reported online education project 347 adolescents. Mean age 16.4 ± 1.12 years (no response rates stated)	April 2019 vs May and June 2020	(1) Positive affect (2) Negative affect (both Positive and Negative Affect Schedule—Children)	Increase in negative affect, decrease in positive affect during the pandemic	(1) Positive affect (2) Negative affect; Before: (1) 3.74 ± 0.61, (2) 2.35 ± 0.70; During: (1) 3.59 ± 0.75, (2) 2.66 ± 0.68; Changes: (1) *p* < 0.001, (2) *p* < 0.001	(1) Positive affect deteriorated (2) Negative affect deteriorated	N/A
Banks and Xu (2020), UK [27]	Self-reported panel data from the nationwide representative UK understanding society household longitudinal study sub-population of 1851 subjects aged 16–24 years (No subpopulation response rates stated)	2009 to 2019 vs April 2020	Psychological distress (General Health Questionnaire-12)	Substantial effects on young adults who already had lower levels of mental health before COVID-19	Psychological distress; Before: 11.83 ± 5.92; During: not available; Changes: original article authors interpretation of significant deterioration among young adults	Psychological distress deteriorated	Gender: significantly worsened psychological distress among young women (16–24) compared to young men and older general population; Existing poor mental health: young women aged 16–24 recorded increasing severe problem
Bignardi et al. (2020), UK [35]	Sample from the Resilience in Education and Development study, from a cohort of children aged 7.6–11.6 years living in the East of England 168 children, mixture of caregiver, teacher and child reports (29% response rate)	June 2018 to September 2019 vs April to June 2020	(1) Depression (2) Anxiety (both Revised Child Anxiety and Depression scale) (3) Emotional problems (Strengths and Difficulties Questionnaire)	Significant increase in depression during pandemic. No significant changes in anxiety and emotional problems subscale	(1)Depression (2) Anxiety (3) Emotional; Before and During: not available; Changes: (1) *p* < 0.001, (2) *p* = 0.035, (3) *p* = 0.173	(1) Depression deteriorated (2) No significant change (anxiety) (3) No significant change (emotional problems)	Age: did not significantly alter the changes; Gender: did not significantly alter the changes
Giménez-Dasí et al. (2020), Italy [26]	Families of preschool and primary school children aged 3.2–11.1 years in two educational centers in northwestern Madrid 113 families, parents’ reports (67% response rate). Mean age 7 years and 2 months ± 2.64 (no response rates stated)	February 2020 vs April 2020	(1) Attentional Problems, (2) Depression, (3) Challenging Behaviors, (4) Emotional Regulation, (5) Hyperactivity, (6) Willingness to study* (System of Evaluation of Children and Adolescents) *(only for the primary education version)	Unchanged for 3-year-old children during pandemic. Lower scores for children aged 6–10 years for attention, emotional regulation, hyperactivity and willingness to study	(1) Attentional problems, (2) Depression, (3) Challenging Behaviors, (4) Emotional regulation, (5) Hyperactivity, (6) Willingness to study; Before: Preschool (1) 2.11 ± 0.68, (2) 1.19 ± 0.24, (3) 2.64 ± 0.69, (4) 2.26 ± 0.67, (5) 2.41 ± 0.77 Primary school (1) 2.21 ± 0.81, (2) 1.40 ± 0.45, (3) 2.30 ± 0.77, (4) 2.15 ± 0.74, (5) 0.71 ± 0.75, (6) 3.02 ± 0.65; During: preschool (1) 2.31 ± 0.76, (2) 1.28 ± 0.45 (3) 2.67 ± 0.83, (4) 2.43 ± 0.92, (5) 2.44 ± 0.87 primary school (1) 2.42 ± 0.83, (2) 1.60 ± 0.64, (3) 2.66 ± 0.89, (4) 2.42 ± 0.93, (5) 0.75 ± 0.75, (6) 2.38 ± 0.74; Changes: preschool: no significant changes for all primary school: (1) *p* = 0.02, (4) *p* = 0.01, (5) *p* < 0.001, (6) *p* < 0.001	Preschool (no significant change for all 5) (1) Attentional Problems, (2) Depression, (3) Challenging Behaviors, (4) Emotional Regulation, (5) Hyperactivity primary school (significant change for 1, 4, 5 and 6, and no significant change for 2 and 3) (1) Attentional problems, (2) Depression, (3) Challenging behaviors, (4) Emotional regulation, (5) Hyperactivity, (6) Willingness to study	Age: no significant difference; Gender: no significant difference
Huckins et al. (2020), USA [25]	Students aged 18–22 from Dartmouth college who participated in the self-report StudentLife study 178 students (no response rates stated)	Academic terms September 2017 until September 2018 vs Winter 2020 academic term	(1) Depression (Patient Health Questionnaire-2) (2) Anxiety (Generalized Anxiety Disorder-2)	Significantly increased symptoms of anxiety and depression	(1) Depression, (2) Anxiety; Before and During: weekly EMA; Changes: (1) *p* < 0.001, (2) *p* < 0.001	(1) Depression deteriorated (2) Anxiety deteriorated	N/A
Li H.Y. et al. (2020), China [31]	Undergraduate students attending Hebei Agricultural University in Baoding, China, who had completed the first wave of the self-reported survey in December 2019 were contacted again 555 students took part at a mean age of 19.6 ± 3.4 years (response rate 89%)	December 2019 vs February 2020	(1) Positive affect (2)Negative affect (both Positive and Negative Affect Schedule) (3) Anxiety and depression (Patient Health Questionnaire-4)	Increases in negative affect and symptoms of anxiety and depression	(1) Positive affect, (2)Negative affect, (3) Anxiety and depression; Before: (1) 3.21 ± 0.79, (2) 2.38 ± 0.79, (3) 0.95 ± 0.65; During: (1) 3.26 ± 0.79, (2) 2.24 ± 0.80, (3) 0.76 ± 0.61; Changes: (1) *p* = 0.107, (2) *p* < 0.001, (3) *p* < 0.001	(1) No significant change (positive affect) (2) Negative affect deteriorated (3) Anxiety and depression Deteriorated	N/A
Li W. et al. (2020), China [30]	Second and third-year students across five disciplines from a university in a southeast city of China took part in this self-reported study the 173 students had a mean age of 19.81 ± 0.98 years (33% response rate)	October 2019 vs January 2020 vs March and April 2020	(1) Depression (2) Anxiety (3) Stress (all Chinese Depression Anxiety Stress Scale)	Stress, anxiety and depression decreased during lockdown, before increasing as restrictions eased	(1) Depression (2) Anxiety (3) Stress; Before: (1) 6.25 ± 6.15, (2) 9.23 ± 6.16, (3) 10.95 ± 7.05; During time 1: (1) 4.99 ± 6.15, (2) 5.09 ± 5.90, (3) 7.11 ± 7.18; During time 2: (1) 6.01 ± 6.10, (2) 6.74 ± 5.97, (3) 8.77 ± 7.04; Changes: (1) *p* < 0.001, *p* < 0.001, (2) *p* < 0.001, *p* < 0.001, (3) *p* < 0.001, *p* < 0.001	(1) Depression deteriorated (2) Anxiety deteriorated (3) Stress deteriorated	N/A
Magson et al. (2020), Australia [39]	Sample comprised adolescent aged 13–16 who lived in an urban area of New South Wales, Australia, who were part of the larger self-reported longitudinal Risks to Adolescent Well-being Project 248 responded at a mean age of 14.4 ± 0.5 years (53% response rate)	Throughout 2019 vs May 2020	(1) Depression (the Short Mood and Feelings Questionnaire—Child version) (2) Anxiety (Spence Children’s Anxiety Scale)	Significant increase in symptoms of depression and anxiety during. pandemic	(1) Depression (2) Anxiety; Before: (1) 3.81 ± 4.31, (2) 4.60 ± 3.74; During: (1) 6.12 ± 6.04, (2) 5.10 ± 4.05; Changes: (1) *p* < 0.001, (2) *p* < 0.001	(1) Depression deteriorated (2) Anxiety deteriorated	Age: no significant difference; Gender: more significant changes among girls
Manjareeka and Pathak (2021), India [34]	First year medical school students aged 18–24 at KIIT University, India 101 took part in a self-reported study at a mean age of 19.7 ± 0.7 years (67% response rate)	February 2020 vs May 2020	Anxiety (state version of State Trait Anxiety Inventory)	Mean anxiety scores were significantly lower before than during the COVID-19 lockdown period	Anxiety; Before: 45.70 ± 11.42; During: 47.97 ± 10.80; Changes : *p* = 0.0394	Anxiety deteriorated	Gender: no significant difference
Munasinghe et al. (2020), Australia [29]	Instagram and Facebook used to recruit adolescents aged 13–19 from general population in Sydney 301 had before and during data (no response rates stated)	18 November 2019 to 22 March 2020 (before) vs after 23 March-19 April 2020	Psychological distress (Kessler Psychological Distress 6-item scale)	Social distancing measures during the pandemic were associated with slightly higher increases in psychological distress	Psychological distress; Before (median interquartile range): 15 (11–20); During (median interquartile range): 15.5 (11–20); Changes: OR = 1.48, 95% CI 95% 0.74–2.95	Psychological distress deteriorated	N/A
Niedzwiedz et al. (2021), UK [24]	Self-reported panel data from the nationwide representative UK understanding society household longitudinal study 5875 adults aged 18–24 took part (no subpopulation response rates stated)	2015–2019 vs COVID-19 wave 24th to 30th April 2020	(1) Psychological distress (General Health Questionnaire-12) (2) Loneliness (1) loneliness item: how often felt lonely in last 4 weeks)	Psychological distress and loneliness increased during the pandemic	(1) Psychological distress, (2) Loneliness; Before (%): (1) N/A (2) 13.3 (95% CI 11.6–15.3); During (%): (1) N/A (2) 20.2 (95% CI 16.0–25.2); Changes: (1)Psychological distress (23% to 40% based on original authors figure), (2) loneliness—both showed significant increase (non-overlapping confidence intervals)	(1) Psychological distress deteriorated (2) Loneliness deteriorated	Age: significantly worsened psychological distress among young people (18–24) compared to older general population
Pierce et al. (2020), UK [28]	Self-reported panel data from the nationwide representative UK Understanding Society Household Longitudinal Study adolescents aged 16–24 took part with 1,543 in COVID-19 2020 wave (no subpopulation response rates stated)	2014 to 2019 vs 23–30 April 2020	Psychological distress (General Health Questionnaire-12)	Significant increase in GHQ-12 scores of individuals aged 16–24 compared to pre-pandemic surveys	Psychological distress; Before (mean and 95% CI): 2014–15: 10.9 (10.6–11.1) 2015–16: 10.8 (10.6–11.0) 2016–17: 11.1 (10.8–11.3) 2017–18: 11.6 (11.3–11.8) 2018–19: 12.0 (11.6–12.5); During (mean and 95% CI): 14.7 (14.1–15.3); Changes: GHQ score pandemic 14.7 95% (14.1–15.3). The increases were significant (as shown by their non-overlapping confidence intervals)	Psychological distress deteriorated	Age: significantly worsened psychological distress among young people (18–24) compared to older general population; Existing poor mental health: steepest increase in the proportion of clinically significant psychological distress among female aged 16–24 with already the highest proportions before the pandemic
Rogers et al.(2020), USA [8]	Project AHEAD, a two-wave self-reported longitudinal study, focused on a stratified random sample of adolescents aged 14–17 drawn from a nationally representative database 407 adolescents. Mean age 15.42 ± 1.16 (no response rates stated)	October 2019 vs April 2020	(1) Depression (Children’s Depression Inventory-short version) (2) Anxiety (Generalized Anxiety Disorder scale) (3) Loneliness (Three-item Loneliness Scale)	Small but significant increases in depressive symptoms, anxiety symptoms and loneliness during the pandemic	(1) Depression, (2) Anxiety, (3) Loneliness; Before: (1) 1.75 ± 0.52, (2) 1.64 ± 0.77, (3) 1.3 ± 0.47; During: (1) 1.84 ± 0.56, (2) 1.85 ± 0.79, (3) 1.44 ± 0.53; Changes: (1) t(406) = 3.88, *p* < .001; Cohen’s d = 0.19, (2) t(406) = 5.92, *p* < .001; Cohen’s d = 0.28, (3) t(406) = 5.52, *p* < .001; Cohen’s d = 0.27	(1) Depression deteriorated (2) Anxiety deteriorated (3) Loneliness deteriorated	Existing mental health: depressive symptoms before strong indicator of depressive symptoms during the pandemic
Thorisdottir et al. (2021), Iceland [41]	Self-report Youth in Iceland school surveys carried out by the Icelandic Centre for Social Research and Analysis targeted all secondary schools students aged 13–18 59,701 adolescents took part (63%-86% response rates)	October/February 2016 and 2018 vs October 2020	(1) Depression (Symptom Checklist-90) (2) Mental well-being (the short Warwick Edinburgh Mental Well-being scale)	Increase in depressive symptoms and worsened mental well-being were observed across all age groups during the pandemic, compared with peers of the same age before COVID-19	(1) Depression, (2) Mental well-being; Before (2016): 13 years (1) 16.52 ± 6.89, (2) 25.21 ± 6.02 14 years (1) 17.36 ± 7.43, (2) 25.04 ± 5.79 15 years (1) 18.11 ± 7.83, (2) 24·73 ± 5.93 16 years (1) 18.23 ± 7·50, (2) 25.62 ± 5.75 17 years (1) 18.94 ± 7.66, (2) 25.68 ± 5.81 18 years (1) 19.09 ± 7.62, (2) 25.96 ± 5.80; During (2020): 13 years (1) 18.72 ± 7.21, (2) 23.13 ± 5.44 14 years (1) 19.40 ± 7.77, (2) 22.86 ± 5.64 15 years (1) 19.41 ± 7.69, (2) 23.01 ± 5.62 16 years (1) 20.65 ± 8.10, (2) 23.92 ± 5.26 17 years (1) 22.52 ± 8.29, (2) 23.37 ± 5.19 18 years (1) 22.41 ± 8.29, (2) 23.66 ± 5.19; Changes: (1) (β 0·57, 95% CI 0·53 to 0·60) (2) (β –0·46, 95% CI − 0·49 to −0·42)	(1) Depression deteriorated (2) Mental well-being deteriorated	Gender: increase in depressive symptoms and worsened mental well-being significantly worse in adolescent girls; Age: increase in depressive symptoms and worsened mental well-being significantly worse among 16–18-year-olds
Wang et al.(2020), China [32]	Non-graduating college students aged 18–22 in a top multidisciplinary and research-oriented university in China took part in a self-report study. From 34 provincial-level administrative units and 260 cities in China 1,172 students (30.84% response rate before)	February 2020 vs March 2020	Anxiety (the Self-Rating Anxiety Scale)	Significantly higher anxiety during the pandemic. Significant differences among all males, females, and students majoring in arts and sciences between the two studies	Anxiety; Before: 40.39 ± 9.98; During: 40.77 ± 10.51; Changes: *p* < 0.05	Anxiety deteriorated	Gender: significant increase in anxiety symptoms among female freshmen but not for male freshmen
Wendel et al. (2020), Canada [37]	Parents from 32 kindergarten classrooms in six schools from a large city in Eastern Canada 113 parents provided date on children aged 4–6 years with a mean age of 4.66 ± 0.54 (no response rates stated)	December 2019 to January 2020 vs May to June 2020	(1) Inattention (2) Hyperactivity/Impulsivity (both The ADHD Rating Scale–5 for children and adolescents, home version)	Children’s inattention and hyperactivity/Impulsivity symptoms increased during the pandemic	(1) Inattention, (2) Hyperactivity/Impulsivity; Before and During: not available changes: (1) *p* = 0.00, (2) *p* = 0.02	(1) Inattention deteriorated (2) Hyperactivity/Impulsivity deteriorated	N/A
Xiang et al. (2020), China [23]	Students aged 6–17 from five schools, selected using cluster sampling from 14 districts in Shanghai, took part in both surveys 2427 school students and/or parents reported (79.8% response rate)	3–21 January 2020 vs 13–23 March 2020	Depression (Children’s Depression Inventory-Short form)	The mean CDI-S score significantly decreased between the period before school closure and when schools were closed during the pandemic	Depression; Before: 4.19 ± 2.82; During:3.90 ± 2.56; Changes: *p* < 0.001	Depression improved	N/A

*N/A* not available

### Study designs and sampling

A longitudinal study design was used in 17 studies [8, 23–32, 34–37, 39, 41] and a repeated cross-sectional study design was used in four studies, with different samples at two time points [7, 33, 38, 40]. A nationally representative, population-based dataset or probability sampling was used in seven studies, with sample sizes ranging from 407 to 59,701 subjects [7, 8, 23, 24, 27, 28, 41]. Probability sampling was used in six studies [7, 8, 23, 24, 27, 28] and one was a nationwide study of all adolescents aged 13–18 years [41], which allowed the findings to be generalized at the population level. Non-representative and non-probability sampling was used in 14 studies, with sample sizes that ranged from 101 to 3886 subjects [25, 26, 29–40].

Three quarters (16/21) of the studies covered subjects 10 years old or more, while only five studies included children below 10 years old: 3–11 years [26], 4–6 years [37], 6–17 years [23], 7–11 years [35] and 7–17 years [7]. The informants were children and/or young people in 16 studies [8, 24, 25, 27–34, 36, 38–41]. Two studies used information from parents [26, 37], two used parents and children or young people [7, 23] and one study used a mixture of parents, teachers and children report [35]. All the studies used same informants in the pre-pandemic and pandemic phases.

### Mental health outcomes

All 21 studies measured mental health outcomes during both pre-pandemic and pandemic time points using the same measures. Depression was assessed in 10 studies [7, 8, 23, 25, 26, 30, 33, 35, 39, 41], anxiety was assessed in 9 studies [7, 8, 25, 30, 32–35, 39] and psychological distress in 4 studies [24, 27–29]. Mental well-being [40, 41], loneliness [8, 24], stress [30, 38], positive and negative affect [31, 36] and hyperactivity/impulsivity [7, 37] were assessed in 2 studies respectively. One study assessed both depression and anxiety as one outcome [31]. Quality of life, emotional symptoms, conduct problems and peer problems were assessed in one study [7].

### Changes in mental health before and during the COVID-19 pandemic

**Fig. 2:**
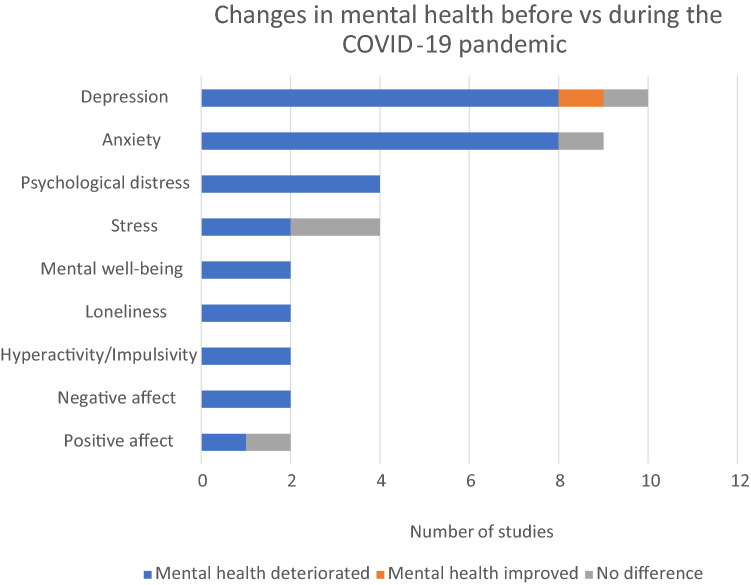
Changes in mental health during the COVID-19 pandemic by the 21 studies based on outcomes (≥ 2 studies)

Most of the studies reported that mental health deteriorated during the COVID-19 pandemic. The findings are summarized in Fig. 2. Increased depressive symptoms were reported by eight studies [8, 25, 26, 30, 33, 35, 39, 41], while one study each reported improve depressive symptoms [23] or no significant difference [7] after the pandemic started. Increased anxiety were reported by eight studies [7, 8, 25, 30, 32–34, 39] while one study found no significant difference [35]. Increased psychological distress was found by all four studies [24, 27–29]. Increased in stress or emotional stress were found in two studies [30, 38], while one study reported no difference in physiological and behavioral stress [38]. Decreased mental well-being was reported by two studies [40, 41]. Two studies reported increased in loneliness [8, 24]. Increases in the negative affect was found in two studies [31, 36], while decreases in positive affects was found in one study [36] and no significant decrease in positive affect in the other study [31].

Mental health change findings for those aged 10–24 years consistently pointed to the deterioration of mental health. We used the World Health Organization definition of children and young people which include adolescents although we noted the arbitrary upper age limit used in some previous studies. The five studies that included children below 10 years old showed varying pattern and mental health outcomes. Out of the 21 included studies, the only study reporting improvement in mental health (depression) symptoms during the pandemic, was a study in China on school children aged 6–17 years [23]. Another study from the UK on those aged 7–12 years found depression symptoms worsened but reported no significant changes for anxiety symptoms and emotional problems [35]. Increased inattention and hyperactivity/impulsivity were found in a study in Canada on young children aged 4–6 years [37]. A repeated cross-sectional study in Germany on children and adolescents aged 7–17 years reported significantly lower health-related quality of life, higher conduct problems, hyperactivity and peer problems during the pandemic, although no significant change for emotional symptoms was reported [7]. A longitudinal study in Italy reported significant deterioration in attentional problems, emotional regulation, hyperactivity, and willingness to study among primary school children aged 6–11.1 years. While similar changes were not shown in any of the outcomes among preschool children aged 3.2–6.2 years [26].

There were few studies assessed the significant influence of of age, gender differences and existing poor mental health when it came to mental health differences before and during the COVID-19 pandemic. Five studies found that mental health symptoms had worsened more in girls than boys [27, 32, 33, 39, 41]. In contrast, one study based on parental reports on adolescents aged 7–17 reported higher increases in mental health problems among boys than girls, in particular in externalizing problems including conduct problems, hyperactivity and peer problems [7]. Two of the largest studies in this review that focused on subjects aged 13–18 [41] and 11–20 [33] years found that mental health problems increased more in older than younger participants during the pandemic. Four studies that looked at subjects aged 16–24 years [27, 40] and 18–24 years [24, 28] compared to subjects older than 24 years old reported higher self-reported psychological distress [24, 27, 28] and higher decreased in mental well-being [40] in these age groups.

Three studies found greater effects in those who already had poor mental health before the COVID-19 pandemic [8, 27, 28]. Young women aged 16–24 who already had poor mental health in the pre-pandemic period, recorded increasing severe problem from 17.7% to 35.2% during the pandemic [27]. Another study showed the steepest increase in the proportion of clinically significant psychological distress in 2020, compared to 2014–2019, among female aged 16–24, the age group with already the highest proportions before the pandemic [28]. One study also showed depressive symptoms before the pandemic were a strong indicator of depressive symptoms among subjects aged 14–17 years during the pandemic [8]. Notably, one study of subjects aged 7–17 years reported that decreases in mental health were most significant in families with low socioeconomic status, migration background and limited living space [7].

## Discussion

To our knowledge, this was the first systematic review to focus on collecting empirical evidence on studies focusing on children and young people aged 0–24 to assess changes in mental health symptoms before and during the COVID-19 pandemic. The studies included more than 96,000 subjects up to 24 years of age before and during the COVID-19 pandemic. Our systematic review of 21 studies from 11 countries had four main findings. First, most of the 21 studies reported a longitudinal deterioration in symptoms for different mental health outcomes especially for adolescents and young people. Second, the overall picture that emerged was that the mental health changes for younger children aged 0–9 years and the influence of gender, age and existing mental issues was limited in our included studies. Third, there was a very limited number of large-scale, repeated cross-sectional designs with clear sampling frames. Fourth, there were also no cross-cultural studies identified that compared how different policies, namely lockdowns, school closures and other social restrictions, may have affected the mental health of the children and young people.

We found that the 21 studies included 23 measures of anxiety, depression or psychological distress and that 20/23 (87%) showed increased levels. There was also some evidence of deteriorated negative affect, mental well-being and increased loneliness during the pandemic. Our review extended the findings from four previous reviews that were largely based on cross-sectional studies [10–13] that showed mental health of children and adolescents may have worsened during the current pandemic. Our findings of deteriorating mental health have global significance, because of the potential long-lasting consequences for the well-being of individuals, families and societies. Numerous birth cohort studies have showed that poor mental health in childhood predicted future mortality and morbidity [42, 43]. In addition, childhood mental health problems have been associated with lower socioeconomic status and less stable social relationships in adulthood [44]. Our previous systematic review showed decreased mental health service use before and during the pandemic in subjects aged 0–24 years during the early phase of COVID-19 pandemic [21]. These two contradictory findings, of increased mental health problems but decreased service use during the early pandemic, indicated unmet needs.

The papers covered by the current review were all based on observational studies and any explanations for why mental health problems increased are largely lacking. Majority of the included studies collected during pandemic data at a very early stage of pandemic, when the more stringent restrictions were largely being implemented globally. A recent longitudinal study on adult population in 15 countries showed that more stringent COVID-19 policies throughout the first 15 month of the pandemic is linked to poorer mental health [45]. Thus, it’s important to note that the present findings reflect the impact during the early phase of the pandemic on the mental health of children and young people. Other studies showed increased online or social media use [46, 47] and problems related to remote learning [48, 49]. These factors need to be explored, together with potential explanations like social isolation, family dysfunction, restricted leisure time activities, worries about being infected or about friends and family being infected and grief at losing loved ones.

Our systematic review showed that few studies have assessed the influence of demographic factors in association with the changes in mental health before and during the pandemic. Given that limited studies have assessed these factors and the variability of outcome in these studies, these results were inconclusive, but mainly in line with previous reviews [9, 11, 12]. Girls and older adolescents were associated with higher deteroriation in mental health symptoms during the pandemic. Mental health symptoms rose more strongly among female than their male peers and this may suggest that they might have been more vulnerable to the psychological effects of the pandemic. Depression and anxiety are increasing during puberty especially among girls [50, 51], which may be one possible explanation for this gender differences. Of note, a previous review showed that the COVID-19 lockdown may have worsened eating disorders symptoms, which are much more common in female [52]. Also, subjects were significantly more affected if they had a pre-existing mental problem [8, 27, 28], low socioeconomic status, migration background and limited living space [7]. More robust research are needed to replicate these findings.

This review has several implications for the methods that should be used by future studies. First, we only found a limited number of studies with large-scale, repeated cross-sectional designs and clear sampling frames. Collishaw’s criteria on time trend studies emphasize the need for comparable measures, clear sampling frames, convergent data by multiple informants and multiple data time points [14]. These kind of study designs are needed for future research, especially as the pandemic remains a global situation. It is possible that the psychological well-being of children and young people will be even more severely affected after longer exposure to the stressors associated with the pandemic. It has been argued that ongoing social isolation, family financial difficulties, missed milestones and school disruption, due to COVID 19 pandemic, will have cumulative adverse effects on the mental health of young people [11]. Second, there were no cross-cultural studies and these are needed to examine how different policies related to school closures and other restrictions have affected child mental health. Research that focuses on the impact of different policies will help societies to be more prepared to address new crises in a more effective and research-based way, such as future pandemics, threats of war and environmental catastrophes. Finally, rigorous studies that identify risk and resilience factors in children and young people during global crises are still lacking. These are urgently needed, so that effective health promotion and prevention strategies can be developed that maintain good mental health in challenging situations.

### Strengths and limitations

The strengths of the study included the fact that the 21 papers we reviewed covered more than 96,000 children and young people aged from 3 to 24 years in 11 countries. These papers provided data on a range of mental health issues and indicated changes in mental health by comparing pre-pandemic and pandemic data. The review also had a number of limitations. We chose not to conduct a statistical meta-analysis, because of the heterogeneity of the study designs and mental health outcomes used by the studies we reviewed. In addition, only peer-reviewed papers published in English were included and we may have missed studies in other languages. Although 21 studies were included, only seven studies had a representative population-based dataset and we were unable to assess mental health on a larger scale. There was also lack of studies on children below 10 years old, limiting the interpretation that can be drawn for this age group. Finally, all the studies that we reviewed focused on changes during the early phase of the pandemic. Future research is needed to see whether subsequent waves had a different impact on the mental health of children and young people.

## Conclusions

This systematic review of 21 peer-reviewed papers showed that the COVID-19 pandemic may have decreased the mental health of adolescents and young people in 11 countries. Future studies are needed to determine the long-term impact of the pandemic on their well-being especially on younger children. It is likely that the world will face similar threats in the future and that these could have an impact on mental health, including new pandemics, wars or environmental catastrophes. Therefore, research on how the COVID-19 pandemic, and different isolation, lockdowns and school closures policies, have affected families, children and young people in different countries, is vital if we are to build future sustainable societies.

## Supplementary Information

Below is the link to the electronic supplementary material.

Supplementary file1 (DOCX 20 KB)

## Data Availability

Not applicable.
