# Supplementary material for: A systematic review of the mental health changes of children and young people before and during the COVID-19 pandemic
Source: Eur Child Adolesc Psychiatry. 2022 Aug 12;32(6):995–1013. doi: 10.1007/s00787-022-02060-0 (PMC9373888; doi:10.1007/s00787-022-02060-0)
Supplement: Supplementary file 1 — Supplementary file1 (DOCX 20 KB) [file 787_2022_2060_MOESM1_ESM.docx]

**Supplementary Appendix**

**Table S1**. Syntax used for the four database searches

**Web of Science (1 January 2020 to 22 March 2021)**

| **Themes** | **Search string** |
| --- | --- |
| **COVID** | TS=(covid* OR coronavirus* OR nCOV OR “SARS-CoV-2” OR quarant* OR lockdown OR pandemic* OR outbreak) AND |
| **Service use or mental health** | TS=(psychiatr* OR psycholog* OR neuropsych* OR mental* OR psychopatholog* OR depress* OR anxi* OR phobia* OR autis* OR ASD OR “attention deficit*” OR stress* OR “posttraumatic stress” OR PTSD OR wellbeing OR well-being OR “quality of life” OR mood* OR insomnia OR suicid* OR “self-harm” OR “self-injury” OR externali* OR internali* OR service* OR admit* OR admission*) AND |
| **Children or Adolescent** | TS=(Infant* OR Child* OR Toddler* OR Adolescen* OR Teen* OR Paediatric* OR Pediatric* OR School* OR “young adult*” OR “young people” OR “young person” OR preteen OR youth* OR student*) AND |
| **Before and during** | TS=(“time trend” OR “time-trend” OR trend OR trends OR “before and during” OR “before and after” OR “cross-sectional” OR longitudinal OR panel OR cohort OR epidemiolog* OR compar* OR differen* OR change* OR before OR registry OR register*) |
| **Total results** | **1652** |

**PubMed (1 January 2020 to 22 March 2021)**

| **Themes** | **Search string** |
| --- | --- |
| **COVID** | "COVID-19"[Mesh] OR "SARS-CoV-2"[Mesh] OR covid*[tiab] OR coronavirus*[tiab] OR nCOV[tiab] OR SARS-CoV-2[tiab] OR quarant*[tiab] OR lockdown[tiab] OR pandemic*[tiab] OR outbreak[tiab] AND |
| **Service use or mental health** | "Psychiatric Department, Hospital"[Mesh] OR "Hospitals, Psychiatric"[Mesh] OR "Emergency Services, Psychiatric"[Mesh] OR "Psychiatric Rehabilitation"[Mesh] OR "Psychosocial Intervention"[Mesh] OR "Mental Health"[Mesh] OR psychiatr*[tiab] OR psycholog*[tiab] OR neuropsych*[tiab] OR mental*[tiab] OR psychopatholog*[tiab] OR depress*[tiab] OR anxi*[tiab] OR phobia*[tiab] OR autis*[tiab] OR ASD[tiab] OR “attention deficit*”[tiab] OR stress*[tiab] OR "posttraumatic stress"[tiab] OR PTSD[tiab] OR wellbeing[tiab] OR "well-being" [tiab] OR “quality of life”[tiab] OR mood*[tiab] OR insomnia[tiab] OR suicid*[tiab] OR "self-harm" [tiab] OR "self-injury" [tiab] OR externali*[tiab] OR internali*[tiab] OR service*[tiab] OR admit*[tiab] OR admission[tiab] AND |
| **Children or adolescent** | "Child, Preschool"[Mesh] OR "Child"[Mesh] OR "Adolescent"[Mesh] OR "Young Adult"[Mesh] OR Infant*[tiab] OR Child*[tiab] OR Toddler*[tiab] OR Adolescen*[tiab] OR Teen*[tiab] OR Paediatric*[tiab] OR Pediatric*[tiab] OR School*[tiab] OR “young adult*”[tiab] OR “young people”[tiab] OR “young person”[tiab] OR preteen[tiab] OR youth*[tiab] OR student*[tiab] AND |
| **Before and during** | "Epidemiologic Studies"[Mesh] OR “time trend”[tiab] OR "time-trend"[tiab] OR trend[tiab] OR trends[tiab] OR “before and during”[tiab] OR “before and after”[tiab] OR “cross-sectional”[tiab] OR longitudinal[tiab] OR panel[tiab] OR cohort[tiab] OR epidemiolog*[tiab] OR compar*[tiab] OR differen*[tiab] OR change*[tiab] OR before[tiab] OR registry[tiab] OR register*[tiab] |
| **Total results** | **333** |

**Embase (from January 2020 to 22 March 2021)**

| **Themes** | **Search string** |
| --- | --- |
| **COVID** | covid*:ti,ab OR coronavirus*:ti,ab OR nCOV:ti,ab OR SARS-CoV-2:ti,ab OR quarant*:ti,ab OR lockdown:ti,ab OR pandemic*:ti,ab OR outbreak:ti,ab AND |
| **Service use or mental health** | psychiatr*:ti,ab OR psycholog*:ti,ab OR neuropsych*:ti,ab OR mental*:ti,ab OR psychopatholog*:ti,ab OR depress*:ti,ab OR anxi*:ti,ab OR phobia*:ti,ab OR autis*:ti,ab OR ASD:ti,ab OR ‘attention deficit*’:ti,ab OR stress*:ti,ab OR ‘posttraumatic stress’:ti,ab OR PTSD:ti,ab OR wellbeing:ti,ab OR ‘well-being‘:ti,ab OR ‘quality of life’:ti,ab OR mood*:ti,ab OR insomnia:ti,ab OR suicid*:ti,ab OR ‘self-harm‘:ti,ab OR ‘self-injury‘:ti,ab OR externali*:ti,ab OR internali*:ti,ab OR service*:ti,ab OR admit*:ti,ab OR admission:ti,ab AND |
| **Children or adolescent** | Infant*:ti,ab OR Child*:ti,ab OR Toddler*:ti,ab OR Adolescen*:ti,ab OR Teen*:ti,ab OR Paediatric*:ti,ab OR Pediatric*:ti,ab OR School*:ti,ab OR ‘young adult*’:ti,ab OR ‘young people’:ti,ab OR ‘young person’:ti,ab OR preteen:ti,ab OR youth*:ti,ab OR student*:ti,ab AND |
| **Before and during** | ‘time trend’:ti,ab OR ‘time-trend’:ti,ab OR trend:ti,ab OR trends:ti,ab OR ‘before and during’:ti,ab OR ‘before and after’:ti,ab OR ‘cross-sectional’:ti,ab OR longitudinal:ti,ab OR panel:ti,ab OR cohort:ti,ab OR epidemiolog*:ti,ab OR compar*:ti,ab OR differen*:ti,ab OR change*:ti,ab OR before:ti,ab OR registry:ti,ab OR register*:ti,ab |
| **Total results** | **549** |

**PsychINFO (1 January 2020 to 22 March 2021)**

| **Themes** | **Search string** |
| --- | --- |
| **COVID** | TS=(covid* OR coronavirus* OR nCOV OR “SARS-CoV-2” OR quarant* OR lockdown OR pandemic* OR outbreak) AND |
| **Service use or mental health** | TS=(psychiatr* OR psycholog* OR neuropsych* OR mental* OR psychopatholog* OR depress* OR anxi* OR phobia* OR autis* OR ASD OR “attention deficit*” OR stress* OR “posttraumatic stress” OR PTSD OR wellbeing OR well-being OR “quality of life” OR mood* OR insomnia OR suicid* OR “self-harm” OR “self-injury” OR externali* OR internali* OR service* OR admit* OR admission*) AND |
| **Children or adolescent** | TS=(Infant* OR Child* OR Toddler* OR Adolescen* OR Teen* OR Paediatric* OR Pediatric* OR School* OR “young adult*” OR “young people” OR “young person” OR preteen OR youth* OR student*) AND |
| **Before and during** | TS=(“time trend” OR “time-trend” OR trend OR trends OR “before and during” OR “before and after” OR “cross-sectional” OR longitudinal OR panel OR cohort OR epidemiolog* OR compar* OR differen* OR change* OR before OR registry OR register*) |
| **Total results** | **137** |

**Table S2.** NIH quality appraisal

| **Author** | **C1.** | **C2.** | **C3** | **C4.** | **C5.** | **C6.** | **C7.** | **C8.** | **C9.** | **C10.** | **C11.** | **C12.** | **C13.** | **C14.** | **Quality** |
| --- | --- | --- | --- | --- | --- | --- | --- | --- | --- | --- | --- | --- | --- | --- | --- |
| Chen et al.(2021) | Yes | Yes | NR | Yes | Yes | N/A | Yes | N/A | Yes | Yes | Yes | No | N/A | No | Good |
| Gray et al.(2020) | Yes | Yes | CD | Yes | Yes | N/A | Yes | N/A | Yes | Yes | Yes | No | N/A | Yes | Good |
| Liébana-Presa et al.(2020) | Yes | Yes | No | Yes | Yes | N/A | Yes | N/A | Yes | Yes | Yes | No | N/A | No | Fair |
| Ravens et al. (2021) | Yes | Yes | No | Yes | Yes | N/A | Yes | N/A | Yes | Yes | Yes | No | N/A | Yes | Good |
| Alivernini et al.(2021) | Yes | Yes | NR | Yes | No | N/A | Yes | N/A | Yes | Yes | Yes | No | Yes | Yes | Good |
| Banks et al.(2020) | Yes | Yes | CD | Yes | Yes | N/A | Yes | N/A | Yes | Yes | Yes | No | N/A | Yes | Good |
| Bignardi et al.(2020) | Yes | Yes | No | Yes | Yes | N/A | Yes | N/A | Yes | Yes | Yes | No | No | Yes | Fair |
| Giménez-Dasí et al.(2020) | Yes | Yes | NR | Yes | No | N/A | Yes | N/A | Yes | Yes | Yes | No | No | Yes | Fair |
| Huckins et al.(2020) | Yes | Yes | NR | Yes | Yes | N/A | Yes | N/A | Yes | Yes | Yes | No | Yes | Yes | Good |
| Li H et al.(2020) | Yes | Yes | Yes | Yes | Yes | N/A | Yes | N/A | Yes | Yes | Yes | No | Yes | Yes | Good |
| Li W et al.(2020) | Yes | Yes | No | Yes | Yes | N/A | Yes | N/A | Yes | Yes | Yes | No | No | Yes | Fair |
| Magson et al.(2021) | Yes | Yes | Yes | Yes | Yes | N/A | Yes | N/A | Yes | Yes | Yes | No | CD | Yes | Good |
| Manjareeka et al.(2021) | Yes | No | Yes | Yes | No | N/A | Yes | N/A | Yes | Yes | Yes | No | Yes | No | Fair |
| Munasinghe et al (2020) | Yes | Yes | No | Yes | No | N/A | Yes | N/A | Yes | Yes | Yes | No | No | No | Fair |
| Niedzwiedz et al (2021) | Yes | Yes | CD | Yes | Yes | N/A | Yes | N/A | Yes | Yes | Yes | No | N/A | Yes | Good |
| Pierce et al (2020) | Yes | Yes | CD | Yes | Yes | N/A | Yes | N/A | Yes | Yes | Yes | No | N/A | Yes | Good |
| Rogers et al.(2020) | Yes | Yes | NR | Yes | No | N/A | Yes | N/A | Yes | Yes | Yes | No | No | Yes | Fair |
| Thorisdottir et al.(2021) | Yes | Yes | Yes | Yes | Yes | N/A | Yes | N/A | Yes | Yes | Yes | No | N/A | Yes | Good |
| Wang et al.(2020) | Yes | Yes | No | Yes | No | N/A | Yes | N/A | Yes | Yes | Yes | No | No | Yes | Fair |
| Wendel et al.(2020) | Yes | Yes | NR | Yes | No | N/A | Yes | N/A | Yes | Yes | Yes | No | No | Yes | Fair |
| Xiang,et al (2020) | Yes | Yes | Yes | Yes | No | N/A | Yes | N/A | Yes | Yes | Yes | No | Yes | Yes | Good |

NR: not reported, CD: cannot be determined, N/A: not applicable

Table S2. NIH criteria of 21 included studies. Quality was rated as fair (< 80%), or good (80% or above); NR: not reported, CD: cannot be determined, N/A: not applicable. The criteria were: C1. Was the research question or objective in this paper clearly stated? C2. Was the study population clearly specified and defined? C3. Was the participation rate of eligible persons at least 50%? C4. Were all the subjects selected or recruited from the same or similar populations (including the same time period)? Were inclusion and exclusion criteria for being in the study prespecified and applied uniformly to all participants? C5. Was a sample size justification, power description, or variance and effect estimates provided? C6. For the analyses in this paper, were the exposure(s) of interest measured prior to the outcome(s) being measured? C7. Was the timeframe sufficient so that one could reasonably expect to see an association between exposure and outcome if it existed? C8. For exposures that can vary in amount or level, did the study examine different levels of the exposure as related to the outcome (e.g., categories of exposure, or exposure measured as continuous variable)?, C9. Were the exposure measures (independent variables) clearly defined, valid, reliable, and implemented consistently across all study participants? C10. Was the exposure(s) assessed more than once over time? C11. Were the outcome measures (dependent variables) clearly defined, valid, reliable, and implemented consistently across all study participants? C12. Were the outcome assessors blinded to the exposure status of participants? C13. Was loss to follow-up after baseline 20% or less? C14. Were key potential confounding variables measured and adjusted statistically for their impact on the relationship between exposure(s) and outcome(s)?
